# PIN1 regulates epidermal cells development under drought and salt stress using single-cell analysis

**DOI:** 10.3389/fpls.2022.1043204

**Published:** 2022-11-14

**Authors:** George Bawa, Zhixin Liu, Rui Wu, Yaping Zhou, Hao Liu, Susu Sun, Yumeng Liu, Aizhi Qin, Xiaole Yu, Zihao Zhao, Jincheng Yang, Mengke Hu, Xuwu Sun

**Affiliations:** State Key Laboratory of Cotton Biology, Key Laboratory of Plant Stress Biology, School of Life Sciences, Henan University, Kaifeng, China

**Keywords:** abiotic stress, arabidopsis, epidermal cells, leaf development, PINFORMED1

## Abstract

Over the course of evolution, plants have developed plasticity to acclimate to environmental stresses such as drought and salt stress. These plant adaptation measures involve the activation of cascades of molecular networks involved in stress perception, signal transduction and the expression of stress related genes. Here, we investigated the role of the plasma membrane-localized transporter of auxin PINFORMED1 (PIN1) in the regulation of pavement cells (PCs) and guard cells (GCs) development under drought and salt stress conditions. The results showed that drought and salt stress treatment affected the development of PCs and GCs. Further analysis identified the different regulation mechanisms of PIN1 in regulating the developmental patterns of PCs and GCs under drought and salt stress conditions. Drought and salt stress also regulated the expression dynamics of PIN1 in *pif1/3/4/5* quadruple mutants. Collectively, we revealed that PIN1 plays a crucial role in regulating plant epidermal cells development under drought and salt stress conditions, thus contributing to developmental rebustness and plasticity.

## Introduction

As a result of global warming and possible climate anomalies, plants often encounter a high level of biotic and abiotic stresses, which engage them in challenging moments that affect their survival and yield (Ramegowda and Senthil-Kumar, 2015; Wang et al., 2021b). During such conditions, plants tend to fine-tune their developmental process and environmental response *via* changes in several transcriptional and metabolic programs (Sun et al., 2007; Sun et al., 2010; Meng et al., 2016; Bawa et al., 2019; Guo et al., 2019; Tang et al., 2019; Sun et al., 2020; Li et al., 2021; Zhou et al., 2021; Liu et al., 2022b; Mostofa et al., 2022; Wu et al., 2022a; Yeshi et al., 2022). For example, drought and salt stress regulate plant growth and development (Li et al., 2016; Devkar et al., 2020; Zelm et al., 2020; Zhang et al., 2020; Karimi et al., 2021; Zhang et al., 2022), and a large number of plants respond to drought stress *via* reducing water loss through stomatal closure (Daszkowska-Golec and Szarejko, 2013; Pirasteh-Anosheh et al., 2016) as well as decreasing rate of leaf expansion under salt stress condition (Rasool et al., 2013; Muchate et al., 2016; Ullah et al., 2020). Drought and salt stress induce osmotic stress, limiting plant normal functions and developmental process, and tolerance to these stresses by plants is of major concern to current agriculture development. Several studies have identified the effects of drought and salt stress on the development and function of plant leaves (Sinha et al., 2016; Ji et al., 2018; Yang and Guo, 2018; López-Serrano et al., 2019; Devkar et al., 2020; Chowdhury et al., 2021). The plant cell wall promotes or impedes plant growth development depending on its mechanical properties, as it often responds to external stimuli, which regulate cell or tissue morphology (Cosgrove, 2017; Sampathkumar, 2020). Many studies have established the link between the development of leaf epidermal cells and leaf morphology (Ferreira et al., 2017; Fujiwara et al., 2018; Zhu et al., 2019). Leaf epidermis plays several key functions including regulating the exchange of gases, water, and nutrients with the surroundings (Zuch et al., 2021). However, the development and function of leaf epidermal cells, such as pavement cells (PCs) and guard cells (GCs), are sometimes disrupted by external environmental factors (Kebbas et al., 2015; Dong et al., 2018; Liu et al., 2022b).

The role of phytohormone’s response to drought and salt stress has been well studied (Ullah et al., 2018; Jalil and Ansari, 2019; Yu et al., 2020; Rehman et al., 2021), among which auxin is a critical hormone (Pandey et al., 2019; Ribba et al., 2020) in the regulation of plant leaf formation (Reinhardt et al., 2003). Interestingly, in the plant epidermal cells, the plasma membrane-localized transporter of auxin PINFORMED1 (PIN1) is the main efflux transporter of auxin that controls the formation and development of leaves and flowers (Reinhardt et al., 2003; Heisler et al., 2005; Heisler et al., 2010). Also, auxin is involved in the regulation of cell polarization, such as the polar distribution of the auxin efflux PIN1 proteins to the plasma membrane and the regulation of root hair initiation areas in the root epidermal cells (Fischer et al., 2006; Dhonukshe et al., 2008); hence it is not surprising that PIN1 regulates the development of leaves in response to auxin signaling (Li et al., 2011; Adamowski and Friml, 2015; Xiong and Jiao, 2019). While the plasticity of plant morphological traits, such as leaf vein pattern development has been linked with varying auxin distribution resulting from changes in PIN1 protein localization (Dhakal et al., 2021), no mechanism has yet been proposed for the role of PIN1 in PCs and GCs development under drought and salt stress conditions. To study the mechanism of epidermal cells development under drought and salt stress, we previously identified the role of *PHYTOCHROME INTERACTING FACTOR (PIF) 1*, *PIF3*, *PIF4*, and *PIF5* genes in PCs and GCs development under drought and salt stress conditions using single-cell RNA-seq analysis (Wu et al., 2022b). However, the mechanism underlying the role of PIN1 in PCs and GCs development under drought and salt stress conditions required further exploration. In the current study, we investigated the role of PIN1 in epidermal cells’ response to drought and salt stress. These results demonstrate that PIN1 is critical in regulating the developmental response of PCs and GCs to drought and salt stress.

## Materials and methods

### Plant materials and growth conditions

Wild type (WT) Arabidopsis (Arabidopsis thaliana) Columbia ecotype (Col-0) was used in this study. The *pif1 pif3 pif4 pif5* quadruple mutant (*pif1-1*, *pif3-7*, *pif4-2*, *pif5-3*), *PIN1pro*:GUS, *pin1-5*, and *35S::PIN1* were obtained from the Arabidopsis Biological Resource Center (ABRC). All mutants and WT Arabidopsis were grown in an artificial climate chamber under the growth conditions of 21-23 ℃, 100 μmol photons m^-2^s^-1^ (normal light treatment), 16 h light/8 h dark and 60%-70% humidity. For NaCl and Mannitol treatments, the seedlings were grown on 1/2MS medium plates containing 100 mM NaCl or 150 mM Mannitol for 1-7 days.

### GUS staining and histological analysis

Histochemical GUS staining was performed as previously described (Liu et al., 2022b). Samples were fixed in 90% acetone at –20°C, rinsed four times with 0.1M sodium phosphate buffer (pH 7.4), and then incubated in X-Gluc solution [0.1M sodium phosphate (pH 7.4), 3 mM potassium ferricyanide, 0.5 mM potassium ferrocyanide, 0.5 g l^–1^ 5-bromo-4-chloro-3-indolyl-β-d-glucuronide cyclohexilammonium salt] at 37°C. After staining, samples were incubated in methanol to remove chlorophyll and then mounted in the clearing solution (a mixture of chloral hydrate, water, and glycerol in a ratio of 8:2:1). Observation was performed using a stereomicroscope (MZ16F, Leica Microsystems, Germany) or a microscope equipped with Nomarski optics (BX51, Olympus Co., Tokyo, Japan).

### Confocal microscopy

The seedlings were stained with 10 g/mL Propidium (PI) (Sigma) for 1 min before imaging. For confocal microscopy, fluorescence in roots was detected using a confocal laser scanning microscope (Zeiss, LSM980). PI signal was visualized using wavelengths of 610 to 630 nm.

### Identification of the genes highly expressed in the corresponding cell type

The average expression and dispersion were briefly calculated for all genes, which were subsequently placed into 9 bins based on expression. Principal component analysis (PCA) was performed to reduce the dimensionality on the log-transformed gene-barcode matrices of the most variable genes. Cells were clustered *via* a graph-based approach and visualized in two dimensions using t-distributed stochastic neighbor embedding (tSNE). A likelihood ratio test, which simultaneously tests for changes in mean expression and percentage of cells expressing a gene, was used to identify significantly differentially expressed genes (DEGs) between clusters. We used the FindAllMarkers function (test.use = bimod, logfc.thresold = 0, min.pct = 0.25) in Seurat to identify DEGs of each cluster. For a given cluster, FindAllMarkers identified positive markers compared with all other cells.

### Total RNA extraction and qPCR analysis

Total RNA was extracted with FastPure Plant Total RNA Extraction kit (Cat. No. DC104, Vazyme; Nanjing, China). Total RNA was treated with DNaseI (Vazyme; Nanjing, China) for 30 min to remove the remaining DNA; then the cDNA was synthesized with HiScript II One-Step RT-PCR Kit (Cat. No. P611, Vazyme; Nanjing, China); qRT-PCR was performed with the corresponding primers (**Supplemental Table 1**). qPCR run was performed on a CFX 96 (Bio-Rad, Herculesm, CA, USA) with the following cycle parameter: 95°C for 30 s, 35 cycles of 95°C for 30 s, 55–56°C for 15 s and 72°C for 15s.

## Results

### Analysis of the expression of PIN1 in different cell types by scRNA-seq

**Figure 1:**
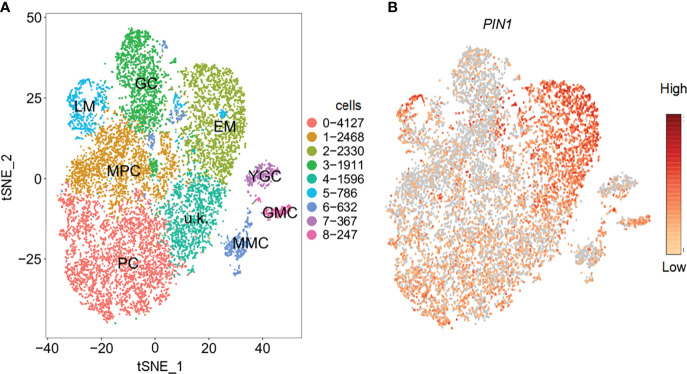
Identification of cell types with representative marker genes. **(A)** Distribution of cell types on tSNE plot; corresponding cell types are annotated on the plot. **(B)** Feature plot showing the expression pattern of *PIN1.* PC, pavement cell; GMC, guard mother cell; GC, guard cell; MMC, meristemoid mother cell; EM, early stage meristemoid; LM, late stage meristemoid; YGC, young guard cell; MPC, mesophyll cell; u.k., unknown.

To determine the possible regulators of PCs and GCs development under drought and salt stress, we first determined the cell types based on previously produced scRNA-seq data (Wu et al., 2022b). The following cell types, PC, guard mother cell (GMC), GC, meristemoid mother cell (MMC), early stage meristemoid (EM), late stage meristemoid (LM), young guard cell (YGC), and mesophyll cell (MPC) were identified based on the known marker genes for the corresponding cell type. A cell cluster without a known marker gene was annotated as unknown (u.k.). We screened the DEGs in the corresponding cell types. During the analysis of the expression patterns of the DEGs, we found that *PIN1* was highly expressed in PC and stomatal lineage cell populations, such as MPC, LM, and EM (**Figure 1**), indicating PIN1’s role in the development of PCs and GCs.

### Detection of the specific expression of pif1/3/4/5 in epidermal cells

**Figure 2:**
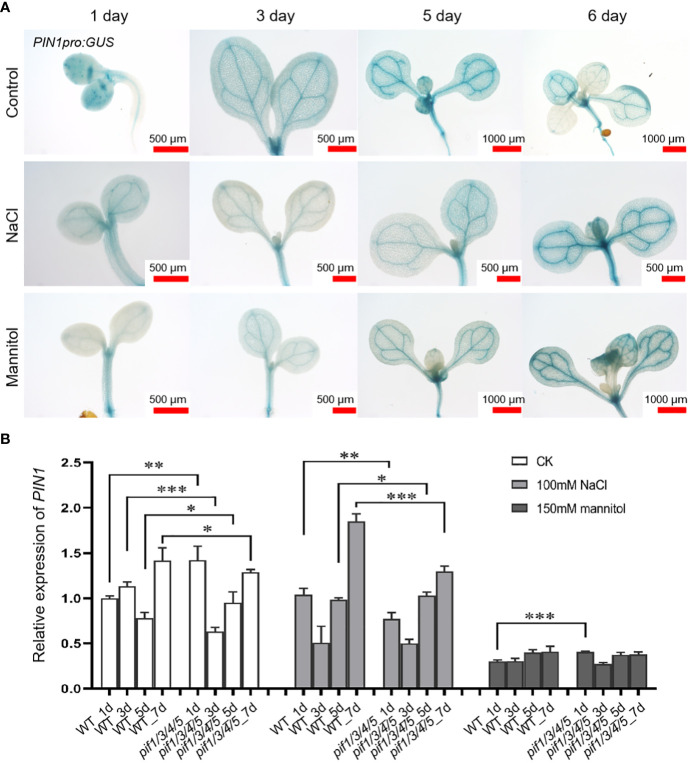
Analysis of the expression of *PIN1pro: GUS* and PIN1 gene after being treated with NaCl and mannitol. **(A)** Analysis of the expression of *PIN1pro: GUS* in whole cotyledons (up panel) during the development of seedlings grown under control, NaCl, and mannitol conditions, respectively. The seedlings of *PIN1pro:GUS* were grown in 1/2 MS plates plus 100 mM NaCl or 150 mM mannitol for 1, 3, 5, and 6 days, respectively; the seedlings of *PIN1pro:GUS* grown in normal 1/2 MS plates were used as controls. Then the seedlings were harvested and used for detecting the GUS activities by GUS staining, as described in Materials and Methods. **(B)** qPCR analysis of the relative expression of *PIN1* under control, NaCl, and mannitol conditions, respectively. Relative expression indicates the mean value ( ± SD) of three independent experiments. The black stars represent student’s t-test of pif1/3/4/5 vs WT. *p < 0.05; **p < 0.01; ***p < 0.001.

In order to analyze the temporal and spatial expression dynamics of *PIN1* under normal, drought, and salt treatment conditions, we used transgenic plants expressing *PIN1pro:GUS* and observed the expression changes of *PIN1* at day 1, 3, 5 and 6 by GUS staining. As shown in **Figure 2A**, under normal growth conditions, there was no significant change in the expression level of *PIN1pro:GUS* in whole cotyledons from day 1 to day 6. Under NaCl treatment conditions, compared with the control, the GUS signal of *PIN1pro: GUS* in whole cotyledon was significantly decreased on day 5, and there was no significant change on the sixth day. Under mannitol treatment conditions, the GUS signal of *PIN1pro:GUS* increased gradually with treatment time. Compared with the control group, the GUS signal of *PIN1pro:GUS* in the cotyledons of day 5 was significantly decreased, and the GUS signal of *PIN1pro:GUS* in the cotyledons of day 6 was not significantly changed, while the GUS signal of *PIN1pro:GUS* was significantly enhanced in the veins of the cotyledons (**Figure 2A**). These results indicated that NaCl and mannitol treatment inhibited the expression of *PIN1pro:GUS* in cotyledons on day 5 before germination and promoted the expression of *PIN1pro:GUS* in vein (**Figure 2A**).

The expression of *PIN1* in *pif1/3/4/5* quadruple mutant under NaCl and mannitol treatment conditions was analyzed by q-PCR. Compared with the control group, NaCl treatment significantly down-regulated the expression of *PIN1* in *pif1/3/4/5* quadruple mutant, but there was no significant change in WT plants (**Figure 2B**). At the same time, mannitol treatment also reduced *PIN1* expression in WT and *pif1/3/4/5* quadruple mutant compared with the control group (**Figure 2B**). Under mannitol treatment conditions, the levels of *PIN1* in *pif1/3/4/5* quadruple mutant on day 1 were significantly higher than WT (**Figure 2B**). Over time, the levels of *PIN1* in *pif1/3/4/5* quadruple mutant were gradually lower than WT (**Figure 2B**).

### Drought and salt stress affect the development of leaf morphology

**Figure 3:**
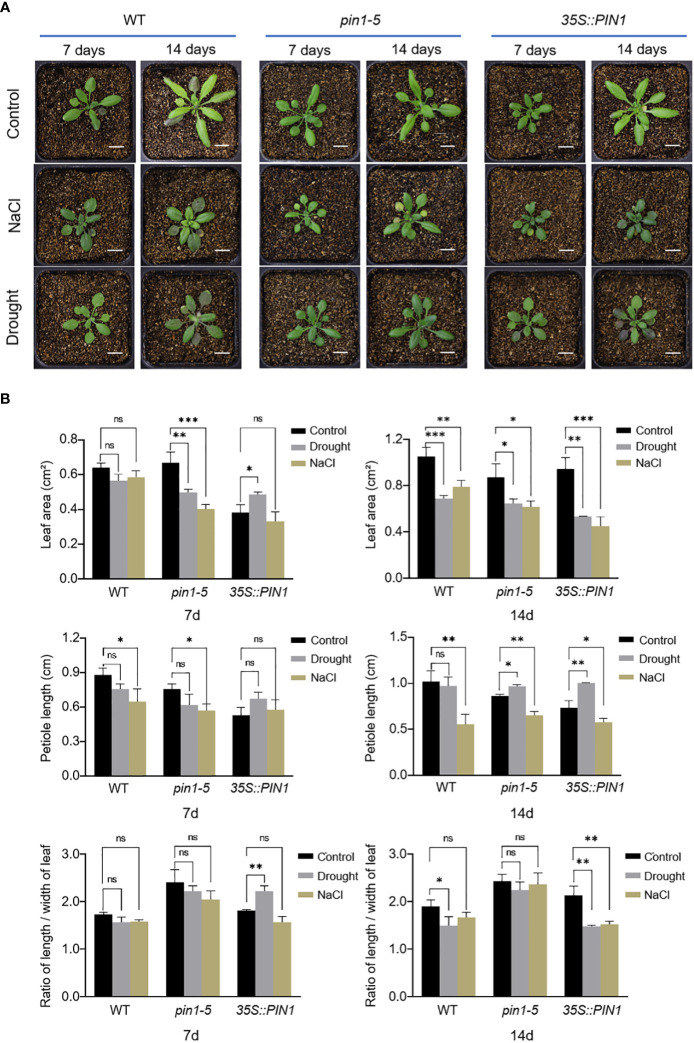
The leaves’ shape changes in response to NaCl and drought stresses. **(A)** *pin1-5*, *35S::PIN1* and WT were grown in soil for drought and NaCl treatment for 7 days and 14 days, respectively; the growth phenotypes were recorded by a camera; untreated seedlings were used as controls. Scale bar: 1 cm. **(B)** Statistical analysis of leaf area, petiole length and ratio of length/width of leaf in *pin1-5*, *35S::PIN1* and WT seeding. The data were analyzed by one-way ANOVA following Brown–Forsythe test. ns: p > 0.05; *p < 0.05; **p < 0.01; ***p < 0.001.

In order to analyze the potential role of PIN1 in regulating plant leaves under drought and salt stress, we analyzed the development of seedlings of *pin1-5* mutant and *35S::PIN1*, the WT was used as control. As shown in **Figure 3A**, compared with WT, *pin1-5* mutant seedlings under normal growth conditions had significantly shorter petioles and a higher leaf length–width (L–W) ratio. Under normal conditions, the leaf development of *35S::PIN1* seedlings was slow in the early stage and faster in the late stage. Between days 7 and 14 after treatment, the leaf area of WT seedlings increased 2 times; the *pin1-5* mutant increased 1.2 times, while the *35S::PIN1* increased 4.5 times. Under NaCl treatment conditions, petiole length of WT was significantly shortened, and L-W ratio of leaves was also decreased (**Figures 3A, B**). Compared with the WT, under drought and NaCl treatment conditions, the leaf area and petiole length of *pin1-5* mutant was significantly shortened, while the ratio of L–W was significantly increased. The petiole length of the *35S::PIN1* was significantly lower than that of WT, while the L–W ratio was significantly higher than that of WT after 7 days of stress treatment (**Figure 3B**).

### PIN1 is involved in regulating the developmental patterns of GC and PC

**Figure 4:**
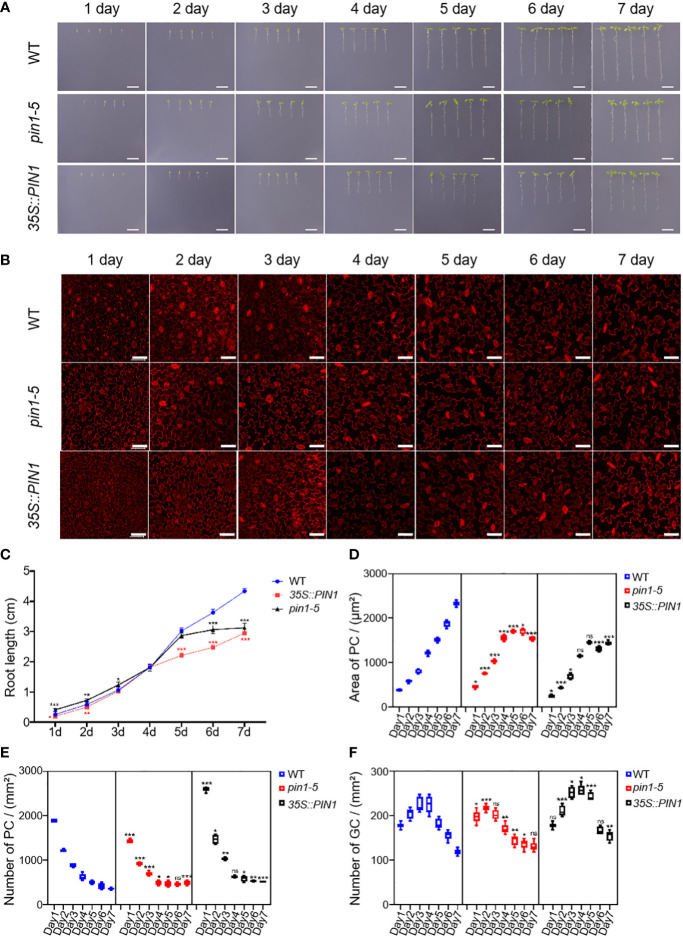
Pavement cells (PCs) and guard cells (GCs) development analysis of *pin1-5*, *35S::PIN1*, and WT under normal conditions. Analysis of the growth of *pin1-5*, *35S::PIN1* and WT under control conditions. **(A)** The seeding of *pin1-5*, *35S::PIN1* and WT were grown on 1/2MS medium for 7 days. The seedlings born 1-7 were photographed continuously by a camera to record their developmental and growth phenotypes. Untreated seedlings were used as controls. Scale bar: 1 cm. **(B)** The development of PC and GC was measured under the following conditions: *pin1-5*, *35S::PIN1* and WT seedlings were cultured in 1/2 MS medium for 1-7 days, then harvested and stained with propyl chloride (PI) for 30 min to stain the membrane. After PI staining, PC and GC′s were detected under a confocal laser microscope. Scale bar: 50 μm. **(C)** Statistical analysis of the root length of seedlings of *pin1-5*, *35S::PIN1* and WT (n = 5). **(D)** Statistical analysis of the size of PCs in the lower epidermis of cotyledons of seedlings of *pin1-5*, *35S::PIN1* and WT (n = 5). **(E)** Statistical analysis of the intensity of PCs in the lower epidermis of cotyledons of seedlings of *pin1-5*, *35S::PIN1* and WT (n = 5). **(F)** Statistical analysis of the intensity of GCs in the lower epidermis of cotyledons of seedlings of *pin1-5*, *35S::PIN1* and WT (n = 5). The data were analyzed by one-way ANOVA following Brown–Forsythe test. ns: p > 0.05; *p < 0.05; **p < 0.01; ***p < 0.001.

To understand the role of PIN1 in the development of PC and GC, we analyzed the developmental dynamics of PC and GC in seedlings of *pin1-5* mutant, *35S::PIN1* and WT during early seedling development. As shown in **Figures 4A, C**, compared with the WT, the growth rate that was characterized by the roots length of *pin1-5* mutant seedlings was faster than that of WT, but the subsequent growth was slower under normal conditions. The germination and subsequent growth of *35S::PIN1* seedlings were slower than that of WT. **Figure 2B** shows the development of PCs and GCs along with the growth time from day 1 to day 7. Statistical analysis showed that in WT, the size of PCs increased rapidly from day 1 to day 7, leading to a gradual decrease in the intensity of PCs (**Figures 4D, E**). Compared with WT, the size of PCs of *pin1-5* mutant showed a similar growth trend to WT from day 1 to day 5, but after day 5, the size of PCs was smaller than WT, resulting in a slightly higher intensity of PCs in *pin1-5* mutant than WT (**Figures 4D, E**). The trend of the size of PCs in *35S::PIN1* was similar to that of *pin1-5* mutant, and the overall size of PCs was smaller than in WT, so the intensity of PCs per unit area was higher than in WT (**Figures 4D, E**). The intensity of GCs per unit area in WT increased rapidly until day 4 before decreasing (**Figure 4F**). Compared with WT, the intensity of GCs per unit area of *pin1-5* mutant was slightly lower, while the intensity of GCs per unit area of *35S::PIN1* was higher (**Figure 4F**).

### Drought and salt stress regulate the developmental patterns of GCs and PCs through PIN1

**Figure 5:**
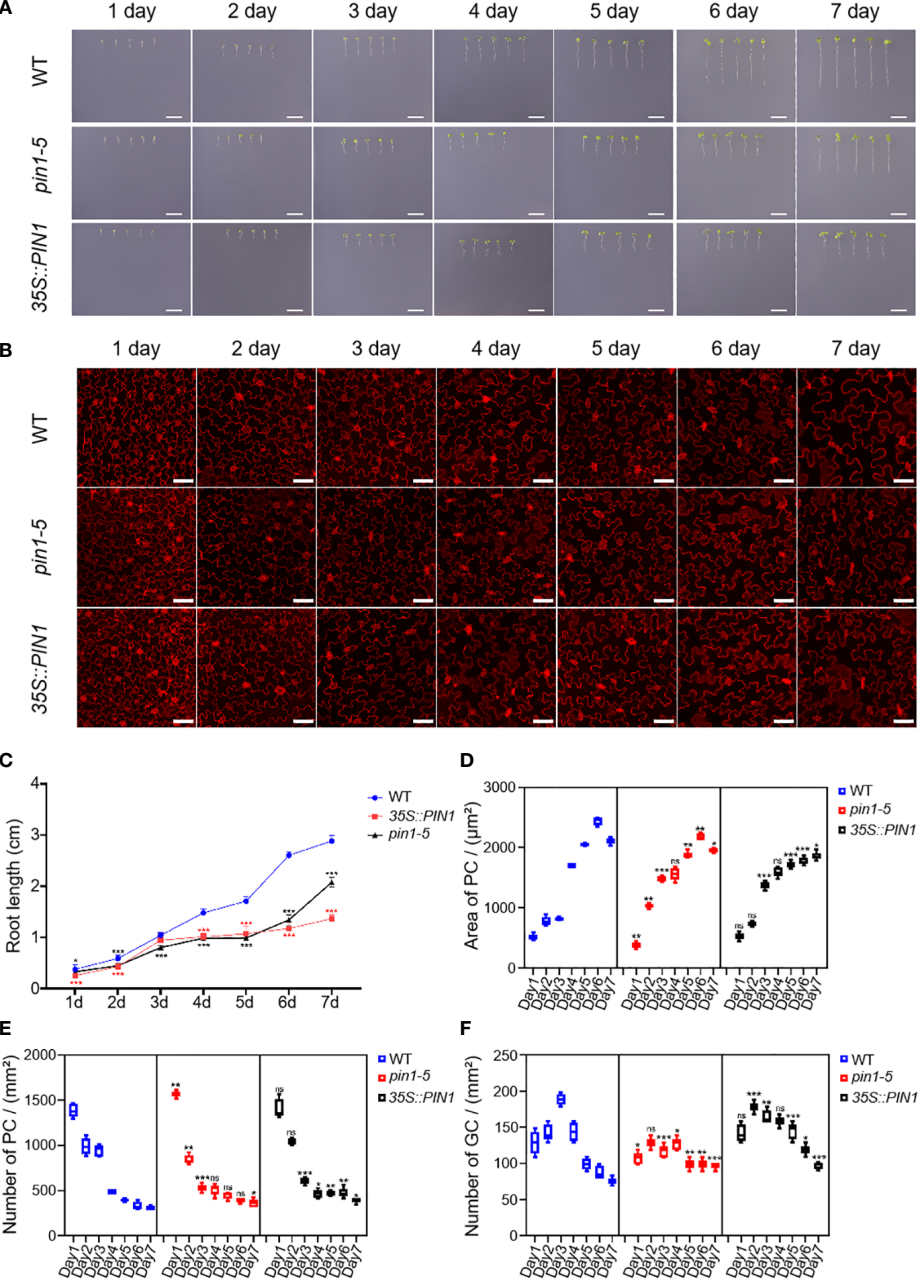
Analysis of PC and GC development in *pin1-5*, *35S::PIN1* and WT under NaCl conditions. Analysis of the growth of *pin1-5*, *35S::PIN1* and WT under control conditions. **(A)** The seeding of *pin1-5*, *35S::PIN1* and WT were grown in 1/2 MS plates plus 100 mM NaCl for 7 days. The seedlings grown for 1~7 days were photographed continuously by a camera to record their developmental and growth phenotypes, and untreated seedlings were used as controls. Scale bar: 1 cm. **(B)** The development of PC and GC was measured under the following conditions: *pin1-5*, *35S::PIN1* and WT seedlings were grown in 1/2 MS plates plus 100 mM NaCl for 1~7 days, then harvested and stained with PI for 30 min to stain the membrane. After PI staining, PC and GC′s were detected under a confocal laser microscope. Scale bar: 50 μm. **(C)** Statistical analysis of the root length of seedlings of *pin1-5*, *35S::PIN1* and WT (n = 5). **(D)** Statistical analysis of the size of PCs in the lower epidermis of cotyledons of seedlings of *pin1-5*, *35S::PIN1* and WT (n = 5). **(E)** Statistical analysis of the intensity of PCs in the lower epidermis of cotyledons of seedlings of *pin1-5*, *35S::PIN1* and WT (n = 5). **(F)** Statistical analysis of the intensity of GCs in the lower epidermis of cotyledons of seedlings of *pin1-5*, *35S::PIN1* and WT (n = 5). The data were analyzed by one-way ANOVA following Brown–Forsythe test. ns: p > 0.05; *p < 0.05; **p < 0.01; ***p < 0.001.

To analyze whether PIN1 was also involved in regulating the differentiation and development of PCs and GCs under NaCl and drought conditions, we analyzed the growth, development and differentiation dynamics of WT, *pin1-5* mutant and *35S::PIN1* seedlings. As shown in **Figures 5A, C**, *pin1-5* mutant and *35S::PIN1* seedlings’ growth was slightly slower than WT from day 1 to day 7 under NaCl treatment conditions. **Figure 5B** shows the developmental dynamics of PCs and GCs in WT, *pin1-5* mutant and *35S::PIN1* seedlings from day 1 to day 7 under NaCl treatment conditions. Statistical analysis showed that under NaCl treatment conditions, the size of PCs in WT seedlings increased gradually from day 1 to day 6 (**Figure 5D**), and the intensity of PCs in WT decreased continuously from day 1 to day 7 (**Figure 5E**). The changing trend of size and intensity of PCs in seedlings of *pin1-5* mutant was similar to that of WT (**Figures 5D, E**). However, the size of PCs in *35S::PIN1* seedlings increased slowly from day 1 to day 7, and was lower than WT (**Figures 5D, E**). The intensity of PCs in *35S::PIN1* seedlings was always higher than WT (**Figures 5D, E**).

Analysis of the intensity of GCs showed that under NaCl treatment conditions, the intensity of GCs in WT increased rapidly from day 1 to day 3 and gradually decreased from day 3 until day 7 (**Figure 5F**). Compared with WT, the intensity of GCs in *pin1-5* mutant seedlings had little change and began to stabilize after day 4. The intensity of GCs in *35S::PIN1* seedlings showed a rapid increase from day 1 to day 2 and then gradually decreased from day 2 to day 7 (**Figure 5F**).

**Figure 6:**
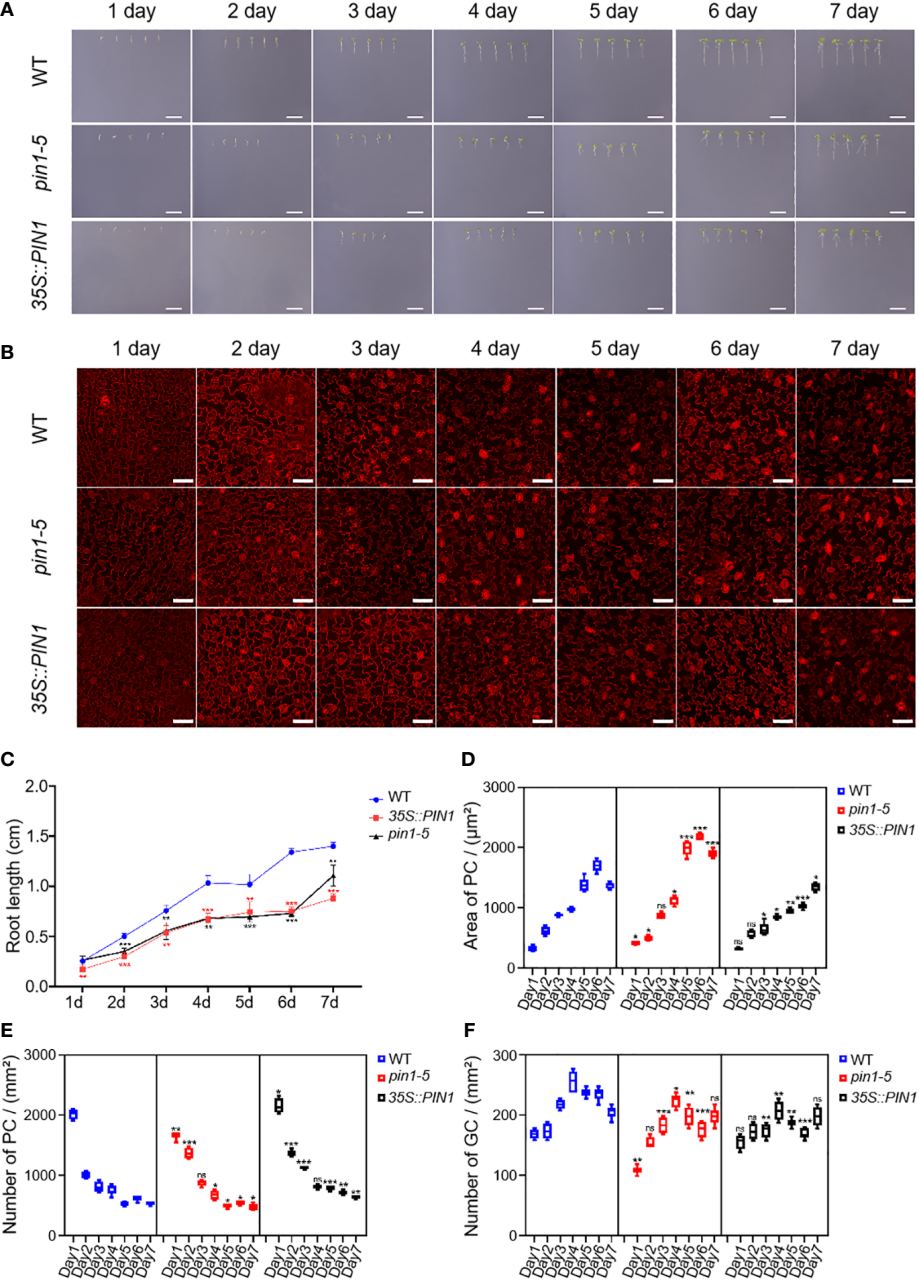
Analysis of PC and GC development in *pin1-5*, *35S::PIN1* and WT under drought conditions. Analysis of the growth of *pin1-5*, *35S::PIN1* and WT under control conditions. **(A)** The seeding of *pin1-5*, *35S::PIN1* and WT were grown in 1/2 MS plates plus 150 mM mannitol for 7 days. The seedlings born 1-7 were photographed continuously by a camera to record their developmental and growth phenotypes, and untreated seedlings were used as controls. Scale bar: 1 cm. **(B)** The development of PC and GC was measured under the following conditions: *pin1-5*, *35S::PIN1* and WT seedlings were grown in 1/2 MS plates plus 150 mM mannitol for 1–7 days, then harvested and stained with PI for 30 min to stain the membrane. After PI staining, PC and GC′s were detected under a confocal laser microscope. Scale bar: 50 μm. **(C)** Statistical analysis of the root length of seedlings of *pin1-5*, *35S::PIN1* and WT (n = 5). **(D)** Statistical analysis of the size of PCs in the lower epidermis of cotyledons of seedlings of *pin1-5*, *35S::PIN1* and WT (n = 5). **(E)** Statistical analysis of the intensity of PCs in the lower epidermis of cotyledons of seedlings of *pin1-5*, *35S::PIN1* and WT (n = 5). **(F)** Statistical analysis of the intensity of GCs in the lower epidermis of cotyledons of seedlings of *pin1-5*, *35S::PIN1* and WT (n = 5). The data were analyzed by one-way ANOVA following Brown–Forsythe test. ns: p > 0.05; *p < 0.05; **p < 0.01; ***p < 0.001.

Compared with the control group, the growth of WT, *pin1-5* mutant, and *35S::PIN1* seedlings was significantly inhibited under mannitol treatment conditions, and the growth rate of *pin1-5* mutant and *35S::PIN1* seedlings was significantly slower than that of WT (**Figures 6A, C**). Compared with WT, under mannitol treatment conditions, the germination rate of *pin1-5* mutant seedlings was faster, but the subsequent growth rate was slower. Both germination and growth of the *35S::PIN1* were inhibited (**Figure 6A**). Under mannitol treatment conditions, the size of PCs in WT seedlings increased gradually from day 1 to day 6 and stabilized on day 7 (**Figure 6D**). The intensity of PCs showed a gradual decline from day 1 to day 7 (**Figure 6E**). Compared with WT, the size of PCs in seedlings of *35S::PIN1* increased gradually from day 1 to day 7 under mannitol treatment conditions, and the changing trend of the size of PCs in seedlings of *pin1-5* mutant was similar to WT (**Figure 6D**). The intensity of PCs in seedlings of WT, *pin1-5* mutant, and *35S::PIN1* showed an opposite trend with the area of PCs (**Figures 6D, E**). Overall, the intensity of PCs in seedlings of *35S::PIN1* was significantly higher than WT, while the intensity of PCs in seedlings of *pin1-5* was lower than WT (**Figure 6E**).

From day 1 to day 4, the intensity of GCs in WT seedlings gradually increased and began to decrease after day 4 (**Figure 6F**). Compared with WT, under mannitol treatment conditions, the intensity of GCs in *pin1-5* mutant and *35S::PIN1* seedlings showed the same trend, gradually increasing from day 1 to day 4 (**Figure 6E**). In general, the intensity of GCs in *pin1-5* mutant and *35S::PIN1* seedlings was significantly lower than that in WT (**Figure 6E**).

**Figure 7:**
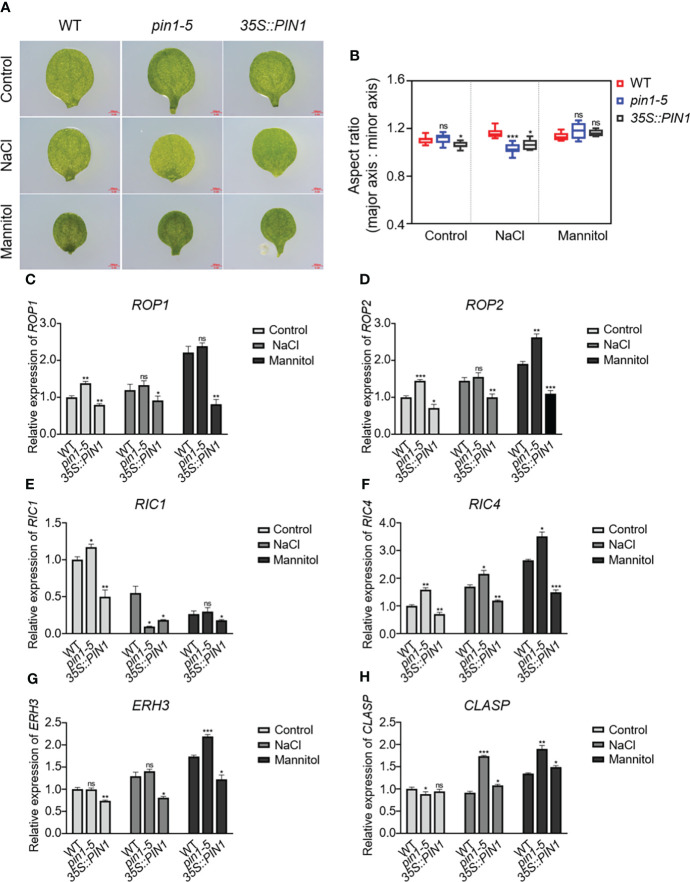
Effects of PIN1 on cotyledon morphological development. **(A)** The wild-type (WT) seedlings, *pin1-5* and *35S::PIN1*, were grown in 1/2 MS plates, 1/2 MS plates plus 100 mM NaCl, and 1/2 MS plates plus 150 mM mannitol for 7 days, and the development of cotyledons was observed on micrographs. Scale bars, 0.5 mm. n ≥ 8 cotyledons per genotype. **(B)** Boxplots represent the aspect ratio of WT and mutant cotyledons. **(C-H)** qPCR analysis of the relative expression of *ROP1*, *ROP2*, *RIC1*, *RIC4*, *ERH3*, and *CLASP* under control, NaCl, and mannitol conditions, respectively. Relative expression indicates the mean value ( ± SD) of three independent experiments. The data were analyzed by one-way ANOVA following Brown–Forsythe test. ns: p > 0.05; *p < 0.05; **p < 0.01; ***p < 0.001.

Considering the defects in stomatal development caused by NaCl and mannitol treatment, it may be that these treatments influenced cotyledon development and altered the expression of *ROP1*, *ROP2*, *RIC1*, *RIC4*, *ERH3* and *CLASP*. To analyze the effects of drought and salt stress on cotyledon development, we analyzed the cotyledon development of WT, *pin1-5* mutant and *35S::PIN1* seedlings under control, NaCl and mannitol treatment conditions (**Figure 7A**). As shown in **Figure 7A**, compared with WT, *35S::PIN1* seedlings under normal growth conditions had significantly reduced aspect ratio relative to wild-type cotyledons, whereas the *pin1-5* mutant remained similar to the wild type. Under NaCl conditions, the cotyledon aspect ratio of WT increased (**Figures 7A, B**). Compared with WT, the cotyledon aspect ratio of *pin1-5* mutant and *35S::PIN1* did not change significantly under mannitol treatment conditions (**Figures 7A, B**). However, under NaCl treatment conditions, the cotyledon aspect ratio of *pin1-5* mutant and *35S::PIN1* was significantly reduced compared with WT. To examine whether PIN could regulate the expression of *ROP1*, *ROP2*, *RIC1*, *RIC4*, *ERH3* and *CLASP*, qPCR analysis was performed. Compared with the control group, NaCl treatment resulted in significantly up-regulated expressions of *ROP1*, *ROP2*, *RIC4* and *ERH3* in WT but not in *pin1-5* mutant seedlings (**Figures 7C, D, F, G**). Meanwhile, mannitol treatment enhanced the expression of *ROP1*, *ROP2*, *RIC4*, and *ERH3* in WT and *pin1-5* mutant compared with the control group (**Figures 7C, D, F, G**). The levels of *ROP1*, *ROP2*, *RIC4* and *ERH3* in the *pin1-5* mutant were also higher than WT under mannitol treatment conditions (**Figures 7C, D, F, G**). For *RIC1*, compared with the control group, its expression was significantly decreased under NaCl and mannitol treatment conditions (**Figure 7E**). As for the expression of *CLASP*, it was found that under NaCl and mannitol treatment conditions, its expression in WT, *pin1-5* mutant, and *35S::PIN1* was significantly increased (**Figure 7H**).

## Discussion

### Drought and salt stresses affect the developmental pattern of leaf epidermal cells

Plants have developed several mechanisms to respond and adapt to different types of abiotic stress enacted by external environmental factors as part of their evolution. How and where plants sense changes in water levels in the root has been well studied. It has been demonstrated that the PLASMA MEMBRANE INTRINSIC PROTEINS (PIPs), a subfamily of plasma membrane-localized aquaporin channels, enhance water movement in plant roots (Dietrich, 2018). Similarly, in salt stress, phosphatidic acid is a minor membrane phospholipid required for plant growth response to salt stress. Phosphatidic acid binds to PINOID (PID) to promote PID-dependent PIN phosphorylation under salt stress (Wang et al., 2019). But the mechanisms underlying how leaf epidermal cells respond to drought and salt stress require further attention. Leaf development can be controlled by multiple regulatory networks. Environmental factors such as drought and salt stress (Chun et al., 2018; Gambetta et al., 2020) affect plants critical developmental processes, such as leaf development. These unfavorable factors invoke changes in plant growth patterns, regulating cell form and function, which plays a role in plant growth style. Although we previously analyzed the role of PHYTOCHROME INTERACTING FACTOR (PIF) 1, PIF3, PIF4, and PIF5 in PCs and GCs development under drought and salt stress (Liu et al., 2022b), no mechanism has yet been proposed for the role of PIN1 in PCs and GCs development under drought and salt stress conditions. Here, we investigated the role of PIN1 in drought and salt stress regulation of the developmental patterns of PCs and GCs in *Arabidopsis* leaves. The data showed that drought and salt stress highly regulated plant leaf morphology *via* decreased leaf area and petiole length in WT, *pin1-5* mutant and *35S::PIN1* seedlings (**Figure 3**). In agreement with these findings, several other studies reported on how drought and salt regulate plant physiology and morphology (Bartels and Sunkar, 2005; Ma and Qin, 2014). The functioning and development of these leaves’ epidermal cells largely depend on each other. More often, the growth and development of the leaf depend on the conditions of these epidermal cells (Bar and Ori, 2014), suggesting that PCs and GCs developmental dynamics are critical for plant response to drought and salt stress conditions.

### PIN1 regulates the development of leaf morphology and epidermal cells under drought and salt stress conditions

Besides biochemical manipulations, mechanical stress induced at the tissue scale influences microtubules to align with the maximal direction of stress, hence regulating cell and tissue level morphogenesis (Hamant et al., 2008; Hervieux et al., 2016; Takatani et al., 2020). Signals from environmental stress induce the expression of temporary regulatory networks, which promotes an overall defense process in plants. A large number of studies have uncovered major genes that regulate leaf epidermal cells development in plants subjected to drought and salt stress (Fricke et al., 2006; Maricle et al., 2009; Taleisnik et al., 2009; Garrido et al., 2014; Zhang et al., 2018; Liu et al., 2022b). This study investigated the role of PIN1 in the development of plant epidermal cells under drought and salt stress. Firstly, we analyzed the developmental dynamics of *pin1-5* mutant, *35S::PIN1* and WT during early seedling development. We observed that the intensity of PCs of *pin1-5* mutant seedlings and *35S::PIN1* seedlings was higher than in WT (**Figure 4**), while the intensity of GCs of *35S::PIN1* seedlings was higher than in *pin1-5* mutant and WT seedlings (**Figure 4**). Our previous findings demonstrated that drought and salt stress regulate the developmental patterns of GCs and PCs (Liu et al., 2022b). Meanwhile, how drought and salt stress regulate the differentiation and developmental patterns of GCs and PCs through PIN1 signaling is not determined yet. In **Figure 5**, we observed that NaCl treatment inhibited the growth of *35S::PIN1* and *pin1-5* mutant seedlings. However, the PC size and number per unit area of *35S::PIN1* seedlings increased more than WT and *pin1-5* mutant seedlings. For GC development under NaCl treatment conditions, we observed that, compared with WT, The intensity of GCs in *35S::PIN1* seedlings showed a rapid increase from day 1 to day 2 and then gradually decreased from day 2 to day 7 (**Figure 3**), indicating that even during growth inhibition by abiotic stress, plants adaptations to cope with and adapt to these stresses have evolved over time, thus why we observed changes in the same organ responding to drought and salt stress, which also suggest that the plasma membrane-localized transporter of auxin PIN1 does not only control the formation and development of flowers and regulation of root hair initiation but also regulate PCs and GCs differentiation and development under salt stress tolerance.

Under mannitol treatment, compared with the control group, the growth of WT, *pin1-5* mutant and *35S::PIN1* seedlings was significantly inhibited. However, compared with WT and *pin1-5* mutant, the size of PCs of *35S::PIN1* seedlings increased. Although the intensity of PCs in WT, *pin1-5* mutant and *35S::PIN1* seedlings showed an opposite trend with the area of PCs, the overall intensity of PCs in *35S::PIN1* seedlings was significantly higher than in WT and *pin1-5* mutant (**Figure 6**). However, the intensity of GCs in *pin1-5* mutant and *35S::PIN1* seedlings was slightly increased, which highlights the involvement of PIN1 in PCs and GCs differentiation and development under drought stress tolerance.

Several genes involved in signaling and regulatory pathways or enzymes known to alleviate plant stress have been reported (Wang et al., 2003; Kim et al., 2010; Huang et al., 2012; Chen et al., 2021; Wang et al., 2021a; Liu et al., 2022a; Liu et al., 2022b). For example, several genes have been involved in the signaling network mediating cell fate determination of the epidermis and stomatal functioning (Pillitteri and Torii, 2012; Mckown and Bergmann, 2020). In addition, since PIN1 expression and distribution in plants is regulated by the auxin signaling pathway (Chen et al., 2011; Omelyanchuk et al., 2016; Guillory and Bonhomme, 2021), it is possible that PIN1 may regulate PCs and GCs development under drought and salt stress *via* auxin signaling. In **Figure 1**, using scRNA-seq analysis, we identified that PIN1 was highly expressed in PCs, suggesting a possible function of PIN1 in regulating PCs and GCs development. For further confirmation, we analyzed the temporal and spatial expression dynamics of PIN1 in *pif1/3/4/5* quadruple mutant under normal, drought, and salt treatment conditions using GUS staining and qPCR analysis (**Figures 2A, B**). The results showed that the expression level of PIN1 in *pif1/3/4/5* quadruple mutant changed under NaCl and mannitol treatments. Compared with the mock, the expression level of PIN1 in *pif1/3/4/5* quadruple mutant was down regulated under NaCl and mannitol treatments (**Figure 2B**). Together, the results demonstrate how NaCl and mannitol treatments regulate the expression of PIN1 in *pif1/3/4/5* quadruple mutant. Because cotyledon development in plants may be regulated by stomatal functioning, we analyzed the effects of drought and salt stress on cotyledon development. As shown in **Figure 7**, we observed that NaCl and mannitol treatments significantly regulated the cotyledon aspect ratio of *pin1-5* mutant and *35S::PIN1* seedlings compared to that of WT. We also determined whether PIN could regulate the expression of *ROP1*, *ROP2*, *RIC1*, *RIC4*, *ERH3* and *CLASP* using qPCR analysis. We found that compared to the control group, NaCl and mannitol treatments upregulated the expression of *ROP1*, *ROP2*, *RIC4* and *ERH3.* Compared with that of WT, the expression of *ROP1*, *ROP2*, *RIC4* and *ERH3* in the seedlings of *pin1-5* mutant was increased, but decreased in *35S::PIN1* seedlings, suggesting that PIN1 may be involved in regulating the cotyledon development by mediating the expression of these genes under drought and salt stress conditions. Collectively, these results suggest that temporally regulatory networks are crucial in leaf epidermal cell development and differentiation under drought and salt stress, among which PIN1 is a critical regulator.

## Conclusion

In summary, PIN1 is critical in developing leaf epidermal cells under drought and salt stress conditions. PIN1 regulated plant morphology under drought and salt stress. PIN1 was constitutively involved in drought and salt stress regulation of PC and GC development. The determination of possible regulators of PC and GC development under drought and salt stress showed that PIN1 was highly expressed in PC and stomatal lineage cell populations, highlighting PIN1 as a critical regulator of PC and GC development under drought and salt stress. The gene expression analysis showed PIN1 expression dynamics in *pif1/3/4/5* quadruple mutant under drought and salt stress. Collectively, this work sheds light on the role of PIN1 in developing PCs and GCs under drought and salt stress, highlighting this gene as a promising candidate for breeding stress-tolerant crops.

## Data availability statement

The original contributions presented in the study are included in the article/**Supplementary material**. Further inquiries can be directed to the corresponding author.

## Author contributions

Conceptualization of the project: XS. Performance of specific experiments: RW, ZL, YZ, HL, SS, YL, AQ, XY, ZZ, JY, MH, and GB. Data analysis and writing of the first draft: XS and GB. Supervision and validation of the manuscript: XS. All authors approved the final version.

## Acknowledgments

We are grateful to the Arabidopsis Biological Resource Center (ABRC) for the Arabidopsis seeds. This research was supported by the National Natural Science Foundation of China (31670233).

## Conflict of interest

The authors declare that the research was conducted in the absence of any commercial or financial relationships that could be construed as a potential conflict of interest.

## Publisher’s note

All claims expressed in this article are solely those of the authors and do not necessarily represent those of their affiliated organizations, or those of the publisher, the editors and the reviewers. Any product that may be evaluated in this article, or claim that may be made by its manufacturer, is not guaranteed or endorsed by the publisher.
