# Supplementary material for: PIN1 regulates epidermal cells development under drought and salt stress using single-cell analysis
Source: Front Plant Sci. 2022 Nov 14;13:1043204. doi: 10.3389/fpls.2022.1043204 (PMC9716655; doi:10.3389/fpls.2022.1043204)
Supplement: Supplementary file 1 [file Table_1.docx]

**Supplementary Material**

**Table S1. List of oligonucleotides used in this study**

| **Gene** | **Name** | **Sequence (5' → 3')** |
| --- | --- | --- |
| qPCR | | |
| *ROP1* | *ROP1s* | ATCCTGGTGCTGTTCCGATT |
|  | *ROP1a* | GTCAAACACCGCCTTCACAT |
| *ROP2* | *ROP2s* | TGGTGCCGTCGGAAAAACTT |
|  | *ROP2a* | CCAGCAGTATCCCACAATCCA |
| *RIC1* | *RIC1s* | AGACATAACCGAAGCGCACA |
|  | *RIC1a* | AACTTCTGTGTCTGGAGCCG |
| *RIC4* | *RIC4s* | TCGCACATCGGTTGGGAAAG |
|  | *RIC4a* | CAAGCAACTCCGGCGGTATG |
| *ERH3* | *ERH3s* | TCATCCGGTGGTGGTCCTAT |
|  | *ERH3a* | CAAGCCCCATCTTGAGGTGA |
| *CLASP* | *CLASPs* | ATGGTGGCCTTGGTTTGTCA |
|  | *CLASPa* | CCCGTTTAGCGGTCAAGGAT |
| *ACTIN* | ACTINs | AACTGGGATGATATGGAGAA |
|  | ACTINa | CCTCCAATCCAGACACTGTA |
